# 纵隔镜在肺癌鉴别诊断和术前分期中的应用价值

**DOI:** 10.3779/j.issn.1009-3419.2014.02.17

**Published:** 2014-02-20

**Authors:** 锋 毛, 良 张, 明辉 蔡, 征平 丁, 阳 申屠

**Affiliations:** 1 200030 上海，上海交通大学附属胸科医院，上海市肺部肿瘤临床医学中心胸外科 Department of Thoracic Cancer, Shanghai Lung Tumor Clinical Medical Center, Shanghai Chest Hospital, Shanghai Jiaotong University, Shanghai 200030, China; 2 130012 长春，吉林省肿瘤医院胸部肿瘤内科 Department of 1st Medicine, Jilin Provincial Tumor Hospital, Changchun 130021, China

**Keywords:** 肺肿瘤, 纵隔肿物, 纵隔镜检查术, 分期, 诊断, Lung neoplasms, Mediastinal mass, Mediastinoscopy, staging, Diagnosis

## Abstract

**背景与目的:**

探讨纵隔镜检查术对纵隔肿物诊断和肺癌鉴别诊断/术前分期的临床应用价值。

**方法:**

共计361例患者纳入研究，其中术前未确诊的纵隔肿物患者162例，疑诊或确诊肺癌并伴有纵隔淋巴结肿大（短径≥1.0 cm）患者199例。所有患者均接受手术，其中经颈纵隔镜检查术（SCM）308例，经胸骨旁纵隔镜检查术（PM）53例。

**结果:**

① 纵隔镜检查术对纵隔肿物诊断的准确性98.11%，敏感性97.62%，特异性100%，阳性预测值100%，阴性预测值91.67%。②纵隔镜检查术对肺癌纵隔淋巴结转移诊断的准确性98.28%，敏感性98.03%，特异性100%，阳性预测值100%，阴性预测值100%。手术相关并发症共计7例，发生率为1.93%。

**结论:**

纵隔镜检查术创伤轻微、安全可靠、取材足量，对纵隔肿物的诊断价值极高，也是肺癌鉴别诊断和术前分期的重要方法和金标准。

## Abstract (en)

**Background and objective:**

To discuss the clinical application of mediastinoscopy in the differential diagnosis and preoperative staging on lung cancer.

**Methods:**

A total of 361 cases of patients were included in the study, of which 162 cases were undiagnosed mediastinal tumor patients before operation, and 199 patients were suspected or diagnosed with lung cancer or mediastinal lymph nodes enlargment(short diameter ≥1.0 cm). All patients underwent surgery, including 308 cases standard cervical mediastinoscopy (SCM), 53 cases parasternal mediastinoscopy (PM).

**Results:**

Taking pathology diagnosis as the gold standard, the mediastinoscopy diagnostic accuracy, sensitivity, specificity, positive predictive value, negative predictive value are 98.11%, 97.62%, 100%, 100%, 91.67% and 98.28%, 98.03%, a 100%, 100%, 100% to mediastinal masses and mediastinal lymph node metastasis of lung cancer. Total seven cases suffered from complications of surgery-related, the complication rate was 1.93 percent (*P* < 0.05).

**Conclusion:**

The trauma of the mediastinoscopy is slight, which is safe, reliable, able to take in sufficient tissue quantities. Mediastinoscopy is highly helpful not only in diagnostic of mediastinal mass, but also in the differential diagnosis of lung cancer, and it's an important method and the gold standard of preoperative staging on lung cancer.

近年，支气管镜超声引导下针吸活检术（EBUS）发展迅速，但由于该技术在活检部位和取材数量等方面的限制，使得纵隔镜检查术依然是公认的纵隔肿物诊断和肺癌外科分期金标准^[1]^。但国内目前缺乏大宗病例的纵隔镜检查术临床应用报告，对该技术在肺癌术前纵隔淋巴分期中的应用较少见诸报道。本文拟系统回顾分析我院部分纵隔镜检查术病例，借以评价其在纵隔肿物诊断和肺癌鉴别/分期中的临床价值。

## 1 资料与方法

### 1.1 一般病例资料

2009年7月至2012年12月，上海市胸科医院共行电视纵隔镜检查术361例，其中男性201例，女性160例；患者年龄18岁-81岁，平均61岁；术前病理明确为肺癌患者59例，其中鳞癌26例，腺癌33例，有其它恶性肿瘤病史者18例（直结肠癌8例，胃癌3例，肝癌1例，肾癌2例，甲状腺癌1例，乳腺癌2例，鼻咽癌1例）。所有患者术前均行血常规、肝肾功能、凝血机制、心电图及肺功能等相关检查化验，无手术禁忌证。住院资料以上海市胸科医院病案室存档病史资料为准。通过SPSS 13.0数据库采集以下数据：住院号、性别、年龄、诊断时年龄、手术日期、手术类型、病理结果。选择纵隔镜检查术的适应证如下：①经支气管镜或经胸肺穿刺病理已确诊为肺癌，但胸部CT显示纵隔淋巴结短径>1 cm或PET/CT纵隔淋巴结阳性显像，为明确纵隔淋巴结病理分期而行手术^[2, 3]^；②胸部CT或PET/CT高度怀疑肺癌且纵隔淋巴结转移但尚无明确病理诊断，为获取病理学诊断并判断分期而行手术^[4, 5]^；③肿大纵隔淋巴结或纵隔肿物，为明确病理诊断而行手术^[6, 7]^。

### 1.2 手术操作过程

经颈纵隔镜检查术（Standard Cervical Mediastinascopy, SCM）：可活检的淋巴结包括#1、#2、#3、#4和#7站，患者仰卧位，头过度后仰伸直颈部，全麻双腔气管插管，按甲状腺手术切口消毒、铺巾，在胸骨上切迹上方约1 cm处作颈部领式切口，长约3 cm，切开皮下组织和颈阔肌，在正中线上分开两侧的颈前肌群，切开气管前筋膜至气管前间隙，用食指沿气管正中线钝性分离，形成人工隧道，沿人工隧道置入纵隔镜，根据术前影像资料重点探查和活检相应区域纵隔淋巴结。胸骨旁纵隔镜（Parasternal Mediastinascopy, PM）：可活检的淋巴结包括#5和#6站，在距左侧胸骨旁2 cm左右的第2或第3肋间做切口，长约3 cm，逐层切开皮肤、皮下组织和肋间肌，置入纵隔镜，探查第#5组、#6站淋巴结或纵隔肿物，直视下多点活检。标本送术中冰冻病理，若冰冻病理无法明确诊断时，重新采样送检。在保证手术安全性的前提下，尽量多处采样以保证足够的标本量和代表性，并备进一步免疫组化和基因突变等检测之需。

### 1.3 统计学方法

采用SPSS 13.0软件进行统计学分析。计数资料采用卡方检验，以*P* < 0.05为有统计学意义。获得纵隔镜检查术的准确性、敏感性、特异性、阳性预测值和阴性预测值等指标。

## 2 结果

### 2.1 临床结果

共计361例患者行纵隔镜检查术，其中经颈308例，经胸骨旁53例。322例患者获取明确病理诊断（具体结果详见表 1），肺癌患者中病理阴性39例，其中36例患者继行肺叶切除及系统性淋巴结清扫，3例患者改行VATS纵隔及肺门淋巴结活检。经颈手术后第1天出院，胸骨旁术后放置胸管引流，术后第1天拔管，第2天出院。出现并发症7例，发生率1.93%，其中2例损伤喉返神经，3例损伤血管，1例伤口感染，1例损破纵隔胸膜。肺癌患者纵隔镜淋巴结活检阴性者同期行肺癌根治术。纵隔肿物患者无论病理如何均为最终结论依据，两者皆以术后石蜡切片病理学作为诊断金标准。

**1:** 纵隔镜检查术的病理学诊断结果 Pathological diagnostic results of mediastinoscopy

Pathology	Mediastinoscopy
Lung cancer	
Squamous cell carcinoma	53
Adenocarcinoma	97
Poorly differentiated carcinoma	15
Small cell carcinoma	28
Large cell carcinoma	3
Carcinoid	1
Adenosquamous carcinoma	2
Mediastinal mass	
Lymphoma	16
Malignant thymoma (C type)	6
Tumors of the reproductive system	5
Sarcoidosis	54
Tuberculosis	19
Benign mediastinal tumor	9
Metastatic tumors	14
Negative	
Lymph node chronic inflammation and fibrous tissue proliferation	39

### 2.2 病理结果

361例纵隔镜检查术的患者所有病理诊断结果详见表 1

### 2.3 诊断效能

纵隔镜检查术的准确性、敏感性、特异性、阳性预测值、阴性预测值详见表 2。纵隔镜对肺癌及常见纵隔肿物的检出率详见表 3。无论对肺癌的病理分期抑或纵隔肿物的诊断，纵隔镜检查术都具有极高的准确性和敏感性，特异性及阳性预测值可达100%且具有较低的假阴性率，对常见的结节病、结核病和淋巴瘤的诊断率则接近100%。

**2:** 纵隔镜的诊断效能 The diagnostic effect of mediastinoscopy

	Mediastinoscopy	
	Lung cancer	Mediastinal mass
Accuracy	98.28%	98.11%
Sensitivity	98.03%	97.62%
Specificity	100%	100%
Positive predictive value	100%	100%
Negative predictive value	87.88%	91.67%

**3:** 纵隔镜对不同类型肺癌和常见纵隔肿物的检出率 The diagnostic effect of mediastinoscopy on deferent type lung cancer and several mediastinal mass

Pathology	Mediastinoscopy	
	+	-
Lung cancer		
NSCLC	156	3
SCLC	28	1
Sarcoidosis	54	2
Tuberculosis	19	0
Lymphoma	16	0

## 3 讨论

随着影像学技术的飞速发展和临床应用的积累，加之社会经济发展全民健康意识提升，纵隔内疾病的检出率明显增加, 但病理诊断仍然无法逾越，单纯的影象学诊断依然有较高的误诊率，制约着临床治疗的实施。

大量临床实践表明，传统的放射学诊断方法（CT, MRI）的诊断效能较低，有较高的假性机率。在肺癌分期中，PET/CT的敏感性、特异性和准确性均高于CT，但依然存在一定的假阳性和假阴性率，还远远不能取代病理学诊断。但对肺癌患者而言，明确纵隔淋巴结有无转移，攸关肺癌病理类型和TNM分期，直接影响治疗方案的确定，对预后判断也有十分重要的价值，纵隔镜检查术对病灶或纵隔淋巴结的直接活检，其重要价值显而易见。众所周知，目前对肺癌的治疗共识包括：小细胞肺癌以放、化疗为主。非小细胞肺癌须根据分期治疗的基本原则实施，N0或N1患者即行肺癌根治术；对已发生纵隔淋巴结转移的N2期患者，建议采用新辅助化疗2个周期后再考虑手术，有研究表明^[8]^，术前纵隔镜检查明确为IIIa（N2）期，适当的术前化疗可使82.9%的患者纵隔淋巴结转阴。也有研究报道^[9]^，术前化疗使46%的患者降期，与非降期患者相比，5年生存率明显提高。因此，术前准确地评估肺癌纵隔淋巴结转移状况，对肺癌的合理规范化治疗及预后具有重要意义。近年发展起来的经超声支气管镜引导下针吸穿刺活检，对肺癌纵隔淋巴结分期和纵隔肿物的诊断有一定的价值^[10-12]^，但由于获取的组织较少，诊断有时较为困难，尤其对于淋巴瘤极难进一步检测分型。而剖胸手术活检相对而言创伤较大，患者往往难以接受，包括胸腔镜手术虽也可应用于纵隔淋巴结活检，但与纵隔镜手术相比较创伤较大，恢复较慢，胸痛更剧，仅在少数纵隔镜无法活检的部位应用。纵隔镜检查术由于可获得较多组织标本，并具有极高的诊断率，在纵隔肿物的诊断和肺癌鉴别诊断和术前分期中仍具备较高的临床应用价值。

常用的纵隔镜检查术包括标准经颈纵隔镜检查术（SCM）和经胸骨旁纵隔镜检查术（PM）。其中SCM主要用于第#2、#3、#4、#7和#10站淋巴结活检, 但主肺动脉窗和主动脉弓旁（即第#5、#6站）淋巴结则为其盲区。而PM可很好地解决上述问题，主要用于第#5、#6站淋巴结活检。据国外大宗临床资料统计，纵隔镜手术并发症通常不超过2.5%，死亡率低于0.5%，主要包括气胸、出血、喉返神经损伤、气管支气管损伤、食管穿孔及偏瘫等^[13, 14]^。本组手术出现并发症7例，发生率1.93%，其中2例损伤喉返神经，3例损伤血管，1例伤口感染，1例损破纵隔胸膜。纵隔镜检查手术创伤小，国外不少医院将将之作为门诊检查，可在局麻下进行^[15, 16]^。但作者认为纵隔镜检查术存有潜在的重大致命性并发症风险，因此在气管插管全身麻醉下手术更为安全，也可以避免患者不必要的精神紧张和基于气管刺激所造成的不适。若术中出现并发症，对全麻患者能够更及时且方便地处理，因而从安全性角度考虑，我们推荐在全麻下行纵隔镜检查术。由于纵隔镜手术可获得较多的组织标本，因此诊断正确率较高，无假阴性率。Toloza等^[17]^报道，2, 173例纵隔镜检查术的确诊率为93.6%，国内波动在85.71%-99%之间^[18, 19]^。本组纵隔镜检查术对肺癌纵隔淋巴结分期的诊断准确率为98.28%，对纵隔肿物诊断的准确率高达98.11%，其中良恶性疾病的鉴别准确率为100%。当然，纵隔镜检查术也有一定的局限性，譬如难以对位于后纵隔和下纵隔的病变进行活检，对此的策略是采用经气管镜超声引导针吸活检或食道超声内镜针吸活检术或电视胸腔镜活检术。对纵隔镜检查术和EBUS的比较研究显示，纵隔镜和EBUS对肺癌具有相似的的术前诊断和分期作用，但对纵隔肿物的诊断效能则以纵隔镜检查术为高^[20]^。

综上所述，纵隔镜检查术仍然是纵隔肿物诊断和肺癌术前纵隔淋巴结分期的金标准，具有很高的临床应用价值，在把握应用指证和技术标准的前提下，值得充分掌握并推广普及。
